# Sleep Characteristics According to Gender and Age Measured by Wrist Actigraphy

**DOI:** 10.3390/ijerph182413213

**Published:** 2021-12-15

**Authors:** Katarína Kováčová, Katarína Stebelová

**Affiliations:** Department of Animal Physiology and Ethology, Faculty of Natural Sciences, Comenius University, Ilkovicova 6, 842 15 Bratislava, Slovakia; kovacova396@uniba.sk

**Keywords:** actigraphy, sleep, gender, age

## Abstract

The sleep/wake rhythm is one of the most important biological rhythms. Quality and duration of sleep change during lifetime. The aim of our study was to determine differences in sleep efficiency, movement, and fragmentation during sleep period between genders and according to age. Sleep period was monitored by wrist actigraphy under home-based conditions. Seventy-four healthy participants—47 women and 27 men participated in the study. The participants were divided by age into groups younger than 40 years and 40 years and older. Women showed lower sleep fragmentation and mobility during sleep compared to men. Younger women showed a higher actual sleep and sleep efficiency compared to older women and younger men. Younger men compared to older men had a significantly lower actual sleep, lower sleep efficiency and significantly more sleep and wake bouts. Our results confirmed differences in sleep parameters between genders and according to age. The best sleep quality was detected in young women, but gender differences were not apparent in elderly participants, suggesting the impact of sex hormones on sleep.

## 1. Introduction

Circadian rhythms are biological rhythms with a period of approximately 24 h. They are controlled by the suprachiasmatic nuclei of the anterior hypothalamus [1]. These rhythms allow organisms to adapt better to changes in the external environment. One of the basic circadian rhythms is the sleep/wake rhythm, which is regulated by circadian and homeostatic processes that interact with each other [2]. Sleep and wakefulness are maintained and controlled by a complex network, with neurotransmitters as the main components of the ascending arousal system. Suppression of the wake-providing system is ensured by inhibitory neurons of the ventrolateral preoptic area that manage sleep [3,4] Sleeping is an essential process for every living species and is characterized by lowering of body movement, reduced breathing and lower sensitivity to stimuli. It consists of two basics stages: non-rapid eye movement (NREM) sleep and rapid eye movement (REM) sleep, which differ from each other and occur in cycles starting with NREM. The average duration of the human sleep cycle is about 90 min. Approximately 3–5 sleep cycles are repeated in a healthy sleep pattern [5,6].

Sleep patterns and sleep duration can be evaluated by subjective and objective methods. The most widely used subjective methods are sleep questionnaires and diaries (e.g., Pittsburgh Sleep Quality Index (PSQI)). Polysomnography (PSG) is the best-known objective method in sleep research. It is a great tool used for recordings of sleep stages, sleep architecture and parameters assessed total sleep time, sleep efficiency, length of wakefulness after sleep onset, and sleep onset latency. It enables electroencephalograms, electrooculograms and electromyograms records to continuous and simultaneous recording of physiologic activity during sleep. PSG also records respiration, and saturation of oxygen to assess sleep apnea [7,8,9]. However, this method requires the lab environment with professional assistants and participants must wear numerous sensors during sleep in a laboratory condition [7,10,11]. Another objective method is actigraphy, which is a widely used alternative to PSG for the identification of sleep phases and sleep parameters that assess the duration (e.g., time in bed or assumed sleep) and quality of sleep (e.g., sleep efficiency or fragmentation index). This method has high accuracy and sensitivity to PSG but lower specificity for detecting wake, since accelerometric devices rely on the body movements to identify wakefulness. This method uses wrist- (or ankle-) worn accelerometers and allows objective, low-cost, long-term measurements of participants with minimal invasiveness in home-based conditions [10,12,13].

The aim of our study was to explore sleep efficiency, movement and fragmentation during sleep period in women and men of different age groups monitored by wrist actigraphy.

## 2. Materials and Methods

### 2.1. Sample Characteristic

Students and employees mostly from Comenius University in Bratislava and their relatives were involved to participate in the study. Participants were recruited through advertisements at Comenius University, per email, personal invitation, or social media. The screening procedure run mostly per email communication or personal interview involving a detailed explanation of the study design and criteria for the study participation. Before any study procedures were performed every participant was informed about the protection of personal data and gave their written informed consent. The study was performed according to the declaration of Helsinki and confirmed by the Ethical Committee of the Faculty of Natural Sciences in Bratislava (ECH19003).

In the study, 82 healthy adult participants met the inclusion criteria and were involved to participate. Eight participants were excluded from further analysis, because of irregular sleep/wake habits or discrepancies between their Actiwatch records and sleep diaries. Data from 74 participants (47 women and 27 men) were analysed. The age of participants was between 19 and 72 years with the mean age 33 years ± 1.8 (SEM) and had a BMI of 18–35 kg/m^2^ with the mean value of 23.1 ± 0.5 kg/m^2^ (SEM). For the further baseline characteristic of the study group divided by age and gender see Table 1. Sleep data were collected during short photoperiods in February to March 2020 before the COVID-19 pandemic restrictions began.

All the participants were healthy sleepers free from any psychological conditions and sleep disorders. They were not treated by medications that influence sleep or behaviour. The use of oral contraceptives was allowed. None of the participants was a shift-worker, heavy smoker, had not travelled across time zones two months before the start of the study, or had reported any sleep problems.

Before the study beginning participants completed the Epworth sleepiness scale (ESS) [14] and Circadian type questionnaire (CTQ) for identification of internal preferences to morningness or eveningness [15,16]. A score greater than 10 on the ESS was exclusionary factor for study participation. Our participants were predominantly neutral chronotypes with mean CTQ score of 54.4 ± 1.6 (SEM).

### 2.2. Procedure

The actigraphy was measured by a wrist worn Actiwatch AW4 (CamNtech, Fenstanton, UK). All the participants were at the beginning personally instructed how to use actiwatch device. Participants were asked to use the marker button to mark bedtime and wakeup time or any special events during sleep. Participants wore an Actiwatch on their non-dominant hand during the whole study except when attending to personal hygiene. They kept a sleep diary relating to sleep start and sleep end and any factors related to sleep problems and disturbances (headache, digestive problems, etc.).

During the study, participants followed their standard daily routine and kept their own sleep/wake regime, naps were not permitted during the day. The data sampling took place in home-base conditions of participants only during working periods. Participants used blue-light screen savers during the evening and night if using their personal technical devices.

Data from actigraphy were collected for a minimum of two and maximum of four consecutive days and nights (mean 2.4 night per participant). We analysed records from 184 nights. Friday/Saturday and Saturday/Sunday nights were excluded from analyses because of possibly different free-days sleep/wake regimes [17]. The data were averaged for each participant and the averages used in the statistical analyses.

### 2.3. Actigraphy and Sleep Analysis

The Actiwatch AW4 is a small, wrist-worn actigraphy device. It contains a piezo-electric accelerometer and records body movements in all directions. The sampling was run for 24 h, including sleep periods. The amplitude of measured activity was sampled 32 times per second and captured the highest amplitude in that second, representing the peak movement intensity of that second. We collected activity data in 60-s epoch intervals. The data were processed using Actiwatch Activity&Sleep Analysis7 software (CamNtech, Fenstanton, UK). The software has a sleep detection algorithm that generates sleep quotas based on actigraphic counts and separates sleep from wakefulness by analysing activity in each epoch compared to surrounding epochs. The epoch was scored as sleep unless the activity exceeded a certain threshold, which was determined by a selected sensitivity. The measurement sensitivity setting was ‘medium’. An activity score in an epoch below a count of 40 was scored as sleep. The data were analysed manually, with correction of sleep start and sleep end according to participants sleep diary or marker. This approach had been used in our previous studies [18,19].

Analyses of the records during sleep periods provided parameters that showed the quality and quantity of the period. We used the following actigraphy-derived sleep parameters: time in bed (Table 1), actual sleep, actual wake, sleep efficiency, sleep bouts, wake bouts, immobile time, moving time, one-min immobility and fragmentation index (Table 2).

### 2.4. Statistical Analyses

The normality of the data distribution was tested according to Shapiro-Wilk test. Differences between women and men were compared statistically using an unpaired Student’s *t*-test, if parametric. Nonparametric data were evaluated using Mann-Whitney U test. Differences between genders and age groups were compared using ANOVA with Fisher LSD post hoc test, if parametric. Nonparametric data were compared using Kruskal–Wallis test with Dunn’s post hoc test. Statistical analysis was performed in STATISTICA 10 statistical software (StatSoft, Inc., Tulsa, OK, USA). Data are presented in boxplot graphs, which present good graphical images of the distribution of the data. Boxplots are constructed from five values: the minimum value, the first quartile, the median, the third quartile and the maximum value.

## 3. Results

**Figure 1:**
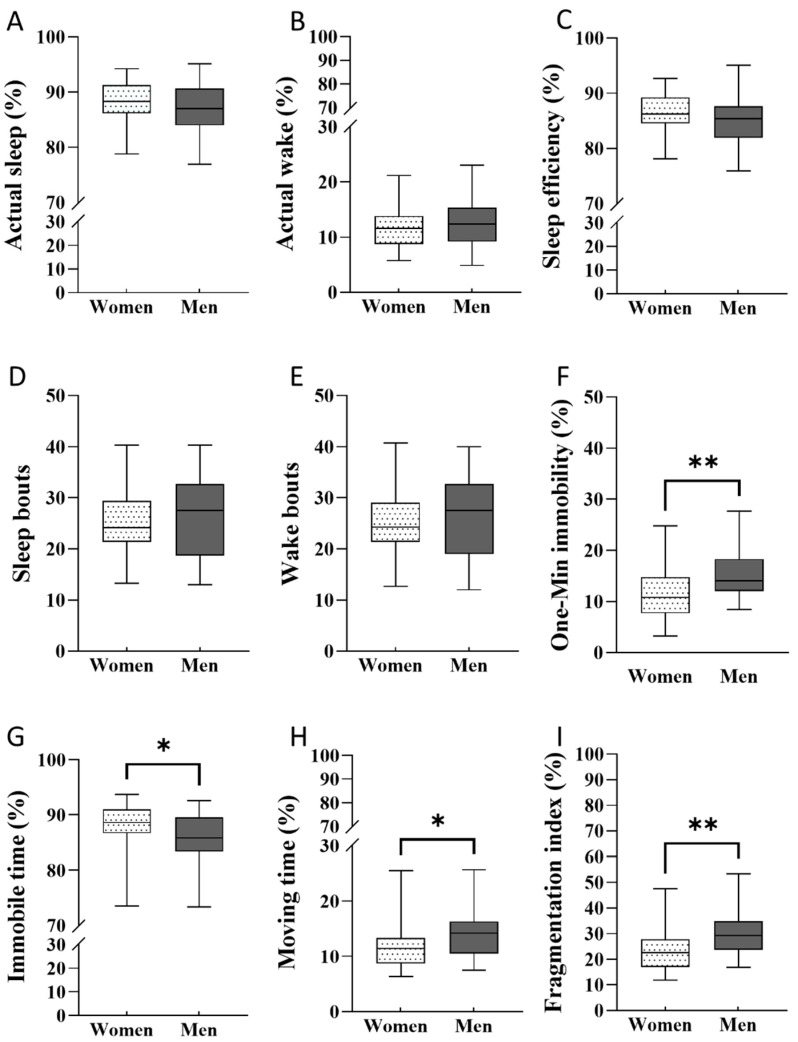
Differences in sleep parameters according to wrist actigraphy between genders. (**A**)—actual sleep (%), (**B**)—actual wake (%), (**C**)—sleep efficiency (%), (**D**)—sleep bouts, (**E**)—wake bouts, (**F**)—one-min immobility (%), (**G**)—immobile time (%), (**H**)—moving time (%), (**I**)—fragmentation index (%). Data are presented as boxplots with median, 25% and 75% quartile and minimum and maximum values for women (*n* = 47) and men (*n* = 27). Statistical differences were compared by unpaired *t*-test, if parametric, or Mann-Whitney U test, if nonparametric (* *p* < 0.05; ** *p* < 0.01).

We found differences in sleep parameters according to actigraphy between women and men (Figure 1). Women’s sleep was significantly less fragmented than was men’s (*p* < 0.01; Figure 1I). Women showed significantly less one-min immobility during sleep compared to men (*p* < 0.01, Figure 1F) and spent a greater percentage of sleeping periods in immobility (*p* < 0.05; Figure 1G) and a significantly lower percentage of time moving during sleep (*p* < 0.05; Figure 1H) compared to men. These differences had no impact on sleep efficiency (Figure 1C), sleep/wake bouts (Figure 1D,E) and actual sleep/wake time (Figure 1A,B).

We divided participants by gender and age into four groups: women younger than 40 years (<40 years; *n* = 35), women 40 years and older (≥40 years; *n* = 12), men younger than 40 years (<40 years; *n* = 17) and men 40 years and older (≥40 years; *n* = 10). The statistical analysis showed significant differences among groups in all tested parameters (Table 3).

**Figure 2:**
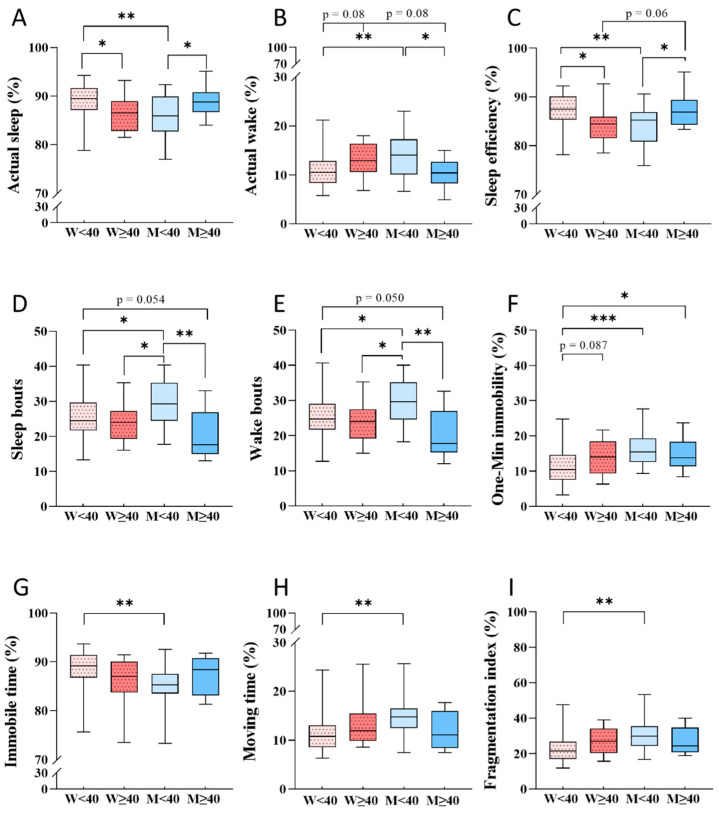
Differences in sleep parameters according to wrist actigraphy according to gender and age: (**A**)—actual sleep (%); (**B**)—actual wake (%); (**C**)—sleep efficiency (%); (**D**)—sleep bouts; (**E**)—wake bouts; (**F**)—one-minute immobility (%); (**G**)—immobile time (%); (**H**)—moving time (%); (**I**)—fragmentation index (%). Data are presented as boxplots with median, 25% and 75% quartile and minimum and maximum values. W < 40—women less than 40 years old (*n* = 35); W ≥ 40—women 40 years and older (*n* = 12); M < 40—men less than 40 years old (*n* = 17); M ≥ 40—men 40 years and older (*n* = 10). Statistical differences were compared by ANOVA with Fisher post hoc test, if parametric, or Kruskal-Wallis with Dunn´s post hoc test, if nonparametric (* *p* < 0.05; ** *p* < 0.01; *** *p* < 0.001).

Post hoc analysis of actual sleep showed that younger group of women (<40 years) have significantly higher percentages of actual sleep compared to more elderly women (≥40 years; *p* < 0.05) and younger men (*p* < 0.01). Younger men (<40 years) showed significantly lower percentages of actual sleep compared to more elderly men (≥40 years; *p* < 0.05; Figure 2A).

The percentage actual wake was lower in younger women than in younger men (*p* < 0.01) and there was tendency to lower actual wake compared to that of more elderly women (*p* = 0.08). Younger men had higher actual wake compared to more elderly men (*p* < 0.05). More elderly women tended to higher actual wake compared to more elderly men (*p* = 0.08) (Figure 2B).

Sleep efficiency in the group of younger women was higher compared to that of more elderly women (*p* < 0.05) and of younger men (*p* < 0.01). On the other hand, sleep efficiency of younger men was significantly lower compared to that of more elderly men (*p* < 0.05). There was a tendency to lower sleep efficiency in the group of more elderly women compared to more elderly men (*p* = 0.06; Figure 2C).

Younger men had significantly more sleep and wake bouts than did younger and more elderly women (*p* < 0.05) and more elderly men (*p* < 0.01). There was tendency to more sleep and wake bouts in the group of younger women compared to the group of more elderly men (*p* = 0.054 and *p* = 0.050, respectively; Figure 2D,E).

Younger women had lower one-min immobility compared to younger men (*p* < 0.001) and more elderly men (*p* < 0.05) and a tendency to lower one-min immobility compared to more elderly women (*p* = 0.087; Figure 2F).

The group of younger women had higher immobile time and shorter moving time compared to the group of younger men (*p* < 0.01 for both parameters; Figure 2G,H).

The fragmentation index for women in the younger group was significantly lower compared to that of younger men (*p* < 0.01; Figure 2I).

## 4. Discussion

Our results showed better sleep quality in women compared to men. These results are in accordance with previous studies [21,22,23,24], in which women showed higher sleep efficiency, higher percentages of deep slow wave sleep stages 3 and 4 and lower percentages of sleep stages 1 and 2 of NREM sleep compared to men [21].

In our study a post hoc sleep analysis showed better sleep in the group of young women compared to the other studied groups. Younger women had much better sleep quality compared to younger men, who had the worst values of sleep parameters of all observed groups. Similar results were found in a study of another authors dealing with university students in which better sleep quality with lower sleep inconsistency observed by accelerometric devices was found in women compared to their male classmates [24]. On the other hand, according to the subjective questionnaire PSQI results, Becker et al. concluded that women compared to men had lower sleep efficiency, more dysfunctions during the day, more sleep disturbances and longer sleep latency. Moreover, women used sleep medications more often than men [25]. These results could also be caused by the different lifestyle of women and men. Various articles and summary studies showed unhealthier lifestyles in men compared to women [26,27,28]. Studies showed that men have higher probabilities of drinking alcohol or smoking and more than 50% of observed men had two or more lifestyle risk factors compared to 20% of women [28,29]. An analysis of dietary-lifestyle patterns and cardiometabolic health reported young men to be a group with unhealthier lifestyles than those of women and more elderly men [30]. The unhealthy lifestyles is possibly leading to a deterioration of sleep quality and could be potential reasons for the observed differences between young women and young men [30,31]. The study of Okano et al., performed using accelerometric devices reported positive correlation between sleep duration and sleep quality among young students, with a stronger correlation in men (r = 0.85) than in women (r = 0.64). A significant negative correlation between sleep inconsistency and sleep quality was found in men but not in women, suggesting a greater importance for men of long-duration sleep and sticking to a regular sleep schedule to get good and adequate sleep [24]. A study observing the inhabitants of Hong Kong by patient-reported outcomes during the social unrest in 2019 showed a higher probability of insomnia in the group of men aged 18–39 compared to women of similar age [32], which may be due to greater resistance to external stressors in younger women [23].

In our study we found no significant differences in sleep between women and men older than 40 years. Women exhibited only a tendency to higher actual wake and lower sleep efficiency compared to more elderly men. Previous studies of other authors showed subjectively lower sleep quality, more awakenings during the night, longer sleep latencies, more frequent use of sedative-hypnotic drugs and disturbed sleep maintenance in more elderly women compared to men of similar age [33]. These results are consistent with another study in which shorter total sleep time, poorer sleep and lower sleep efficiency were reported by women after subjective evaluation. However, actigraphy parameters showed longer sleep, higher sleep efficiency and lower fragmentation index in the group of more elderly women compared to men of similar age [34]. In another study, despite shorter sleep stage NREM1 and longer sleep stage NREM3 in women compared to men, the authors reported higher PSQI score, which means worse sleep, longer sleep latencies, lower sleep efficiencies and daytime dysfunction in women older than 60 years compared to men of the same age group [35]. The authors suggest that these differences can be related to higher relative alpha- and beta-powers observed in women during NREM sleep, a possible higher need for sleep in women and a higher probability of women expressing their emotional distress [34,35].

We found higher percentages of actual sleep and sleep efficiency in young compared to more elderly women, suggesting that sleep in women worsens with age. These findings agree with results of other studies [23,36,37]. Older women in a study by Schwarz et al., showed decreased total sleep time, time spend in third stage of NREM sleep, reduced REM sleep and increased waking after sleep onset in older women compared to men [37]. During ageing, sleep can change due to various physiological and social factors associated with women’s lives [38]. Another studies showed that according to PSQI, the menstrual cycle related to the premenstrual syndrome and dysmenorrhoea affects more than 80% of young women and increases the risk of impaired sleep [39,40]. Moreover, poor sleep and short sleep duration is associated with cycle irregularity and women’s sleep worsens during the menopause, probably due to the withdrawal of oestrogen [23,41]. A study performed on the group of women participants older than 40 years showed a higher probability of insomnia assessed subjectively compared to men of the same age group [32]. Also, meta-analysis showed a higher prevalence of insomnia in women compared to men; this increased with age [42]. Primary insomnia can subsequently lead to shorter total sleep time, reduced sleep efficiency, increased number of awakenings and impairment of sleep architecture [43].

Our observation showed significantly lower sleep efficiency and percentage of actual sleep in younger compared to more elderly men, suggesting worse sleep quality in young men. These findings are inconsistent with those of other studies [44,45]. PSG study of elderly men (51–70 year old) compared to younger men (21–30 year old), observed more time awake, lower sleep efficiency and decreased percentage of time spent in delta sleep, which represents slow-wave sleep in more elderly men [44]. Another study showed higher total sleep time, sleep period time and sleep efficiency index and less waking after sleep onset in young compared to older men. In addition, young men reached persistent sleep and NREM3 sleep earlier and had more slow-wave sleep and less light sleep compared to more elderly men [45]. Study using PSG on almost 3000 men older than 65 years also showed greater percentage of sleep time in NREM1 and NREM2 of NREM sleep and lower percentage in REM sleep in the oldest observed group of men [46]. The worsening of sleep during ageing relates to decreases of sex hormones. This change can have great impact on sleep in women, while the decrease in testosterone is slower and may not have such a large effect on men´s sleep [6,23,47,48]. Although older men in another study had poorer sleep quality and more awakenings during sleep compared to younger men, after sleep deprivation the more elderly participants showed better vigilance, higher alertness and lower sleepiness during the day compared to younger men [49]. Possible sleep deprivation may subsequently lead to greater vulnerability in young compared to older men, which can lead to worse sleep in young men [50].

Our study has several limitations. Despite a relatively large number of participants, the numbers in the groups differed, with the largest group of young women. Due to personal duties of participants, they were not monitored for the same number of days. The length of monitoring may be insufficient for the reliable interpretation of some parameters [51]. Our home-based study provides sleep records from well-known home conditions that could not be precisely controlled.

## 5. Conclusions

We conclude sleep fragmentation and movement differ between men and women according to wrist actigraphy. The best sleep quality with lowest fragmentation of sleep period and highest sleep efficiency was detected in the group of young women. The gender differences disappeared in more elderly women and men, suggesting the impact of sex hormones on sleep.

## Figures and Tables

**Table 1:** Baseline characteristics of participants. Bedtime and Wakeup time—habitual sleep and wakeup times according to sleep diary; Time in bed—time between bedtime and getting up time according to actigraphy; Chronotype score according to CTQ. Data are presented as mean ± SEM for women and men participants divided by age (<40—women/men younger than 40 years; ≥40—women/men 40 years and older).

	Women (*n* = 47)		Men (*n* = 27)	
Group	<40 (*n* = 35)	≥40 (*n* = 12)	<40 (*n* = 17)	≥40 (*n* = 10)
Mean age (years)	22.3 ± 0.5	54.3 ± 2.6	25.4 ± 1.2	54.7 ± 2.2
Age range (years)	19–33	40–72	20–39	44–63
BMI (kg/m^2^)	22.7 ± 0.7	23.5 ± 1	24.4 ± 0.8	25.9 ± 2.2
Bedtime (h:min)	22:24 ± 0:53	22:42 ± 0:18	23:40 ± 0:10	22:20 ± 0:36
Wakeup time (h:min)	7:12 ± 0:11	5:57 ± 0:12	7:51 ± 0:31	5:20 ± 0:20
Time in bed (h:min)	7:35 ± 0:07	7:13 ± 0:12	7:28 ± 0:09	7:12 ± 0:15
Chronotype score	49.9 ± 1.9	63.6 ± 6.1	57.4 ± 2.7	56.9 ± 6.8

**Table 2:** Description of presented sleep parameters according to the Actiwatch user manual V 7.2 and [20].

Sleep Parameter	Description
Actual sleep (%) Actual wake (%)	The time actually spent sleeping or waking, respectively.
Sleep efficiency (%)	Index of the amount of time in bed spent sleeping.
Sleep bouts Wake bouts	The actual number of episodes of sleep and of wakefulness, respectively.
Immobile time (%) Moving time (%)	Percentage value comparing the time spent immobile or moving during the assumed sleep period, respectively.
One-min immobility (%)	The percentage of immobility lasting 1 min or less in relation to the total number of immobility phases.
Fragmentation index (%)	Indicator of restlessness, calculated by the sum of moving minutes and immobility phases shorter than 1 min.

**Table 3:** Statistical comparison of sleep parameters according to gender and age.

Variable	Analysis of Variance	*p*-Value
Actual sleep (%) ^1^	F (3, 71) = 3.600	*p* < 0.05
Actual wake (%) ^1^	F (3, 71) = 3.583	*p* < 0.05
Sleep efficiency (%) ^1^	F (3, 71) = 4.641	*p* < 0.01
Sleep bouts ^1^	F (3, 71) = 4.403	*p* < 0.01
Wake bouts ^1^	F (3, 71) = 4.714	*p* < 0.01
Immobile time (%) ^2^	H (3, 71) = 11.031	*p* < 0.05
Moving time (%) ^2^	H (3, 71) = 10.564	*p* < 0.05
One-min immobility (%) ^1^	F (3, 71) = 4.744	*p* < 0.01
Fragmentation index (%) ^2^	H (3, 71) = 12.542	*p* < 0.01

^1^ ANOVA F-statistic for parametric data distribution; ^2^ Kruskal-Wallis H-statistic for nonparametric data distribution.
